# Quantitative Study of Vitamin K in Plants by Pressurized Liquid Extraction and LC-MS/MS

**DOI:** 10.3390/molecules29184420

**Published:** 2024-09-17

**Authors:** Iryna Bryshten, Łukasz Paprotny, Małgorzata Olszowy-Tomczyk, Dorota Wianowska

**Affiliations:** 1 Department of Chromatography, Institute of Chemical Sciences, Faculty of Chemistry, Maria Curie-Skłodowska University in Lublin, pl. Maria Curie-Skłodowska 3, 20-031 Lublin, Poland; iryna.bryshten@mail.umcs.pl (I.B.); malgorzata.olszowy-tomczyk@mail.umcs.pl (M.O.-T.); 2 Research and Development Centre, ALAB Laboratories, ul. Ceramiczna 1, 20-150 Lublin, Poland; lukasz.paprotny@alab.com.pl

**Keywords:** phylloquinone, vitamin K1, biological activity, green plants, modern extraction techniques

## Abstract

The health-promoting properties of vitamin K stimulate the growing interest in this compound, which translates into the development of new analytical methodologies for its determination. New, more efficient methods of its isolation are sought, paying increasingly more attention to the methods within currently available extraction techniques that, owing to the optimization of the process, not only increase the extraction efficiency but are also economical and environmentally friendly. This article proposes a procedure for the extraction and analysis of one of the vitamin K vitamers, i.e., vitamin K1, using PLE and LC-MS/MS. It has been shown that the PLE technique can be optimized with a mathematical model—accelerating and reducing the costs of the extraction process—which, together with process automation, bodes well for industrial applications. The optimized process was used to extract vitamin K1 from various vegetables, showing very different contents of the test compound ranging from 1.22 to 114.30 µg/g dry weight for avocado and spinach, respectively. In addition, by showing the effect of water within the material subjected to extraction on the variable yield of vitamin K1, attention was drawn to the need to standardize the analytical methods used in assessing the quality of food products.

## 1. Introduction

One of the main problems of the modern world that is observed in many countries is the increase in the average age of people, resulting in a statistically significant increase in the number of diseases related to aging [1,2,3]. These diseases are believed to be lifestyle-related, and eating habits are a significant risk factor for these diseases. With this in mind, current emphasis is placed on broad-based prevention, carried out in cooperation with various environments and institutions, and aimed at improving eating habits and diet.

In light of the recent research results, vitamin K is an important compound that can prevent the appearance and/or development of many age-related diseases, including osteoporosis, circulatory system diseases, and cancer [4,5,6]. It is therefore not surprising that interest in vitamin K is growing, which stimulates the development of new research methodologies [7,8,9,10,11,12,13,14,15,16]. New, more efficient methods of its isolation are sought, paying increasingly more attention to methods within currently available extraction techniques that, owing to process optimization, not only increase the extraction efficiency but also make the process more economical and environmentally friendly [7,17,18,19].

Vitamin K is a family of structurally related compounds. Its main naturally occurring form, characteristic of green plants, is vitamer K1, commonly called phylloquinone [7]. Phylloquinone extraction is most often carried out using the maceration technique at room temperature, after gently heating the extraction system, or it is supported by ultrasound [20,21,22]. Very few papers use modern, more advanced, and assisted extraction techniques that can be used in the industrial isolation of compounds. One such technique is pressurized liquid extraction (PLE). Its essence comes down to the use of elevated pressure to maintain the liquid state of extractants used at temperatures above their boiling points. Therefore, currently, the choice of extraction solvent is not the only key to the success of the isolation process. However, PLE not only uses unusual thermal conditions but also these conditions can be changed across a fairly wide temperature range. Similarly, the pressure conditions and extraction time can be varied. As a result, the PLE technique is considered a fast and effective method for the exhaustive isolation of compounds. It is therefore not surprising that it is currently one of the most popular extraction techniques used for the isolation of both volatile and non-volatile compounds, including biologically active compounds susceptible to the negative impact of high temperatures [23]. In [19], using the example of extraction of the vitamin K1 standard under simulated PLE process conditions, the usefulness of this technique for the isolation of vitamin K1 was also demonstrated; however, the process optimization was neglected.

In the literature, the optimization of the PLE process is mostly performed using the traditional method, i.e., changing only one optimization parameter at a time, such as extraction temperature, type of extractant, extraction pressure, or extraction time. This approach is simple and easy but lengthy. Moreover, due to the lack of optimization for several parameters at once, the interactions between variables are not taken into account, which may significantly modify the extraction efficiency [24]. Therefore, in this study, the PLE extraction conditions were mathematically designed for the optimal efficiency of vitamer K1 isolation from various vegetables and fruits using the central composite design (CCD) method. 

CCD, also called Box–Wilson central composite design, is a type of response surface design that allows for prediction in the design space. It is used when the design plan requires sequential experiments. It is characterized by an imbedded factorial or fractional factorial design with center points, supplemented by a group of axial points. The axial points are popularly called star points and there are always twice as many of them as the factors in the design. They represent new extreme values for each variable in the design and allow for the estimation of curvature [25]. 

In this study, a CCD was constructed to determine the degree of desirability of the response in relation to the intended optimal value, taking into account the variables affecting the extraction efficiency, such as extraction temperature, pressure, and extraction time. The designed optimal extraction conditions were verified by comparing the calculated efficiency with the efficiency obtained during the actual extraction of phylloquinone from iceberg lettuce *(Lactuca sativa* var. *capitata*). Then, the optimized PLE method was used to isolate vitamin K1 from the following plants: cucumber (*Cucumis sativus*), spinach (*Spinacia oleracea*), broccoli (*Brassica oleracea* var. *italica*), bean (*Vicia faba*), bell pepper (*Capsicum annuum*), arugula (*Eruca vesicaria* ssp. *sativa*), kale (*Brassica oleracea*), brussels sprouts (*Brassica oleracea* var. *gemmifera*), chives (*Allium schoenoprasum*), parsley (*Petroselinum crispum*), dill (*Anethum graveolens* L.), and avocado (*Persea americana* Mill.) The developed and optimized ultrasound-assisted solvent extraction (UASE) procedure was used to control the efficiency of the isolation process [26]. The analysis of vitamer K1 content was performed by LC-MS/MS. 

There is not much information in the literature on the content of vitamin K in various food groups, including fruits and vegetables [11,20,21]. Most of the available information is limited to single selected and characteristic regional products [7,10]. This study complements this information by showing in a broader perspective the content of health-valuable vitamin K1 in commonly consumed fruits and vegetables.

## 2. Results and Discussion

### 2.1. LC-MS Procedure

**Figure 1:**
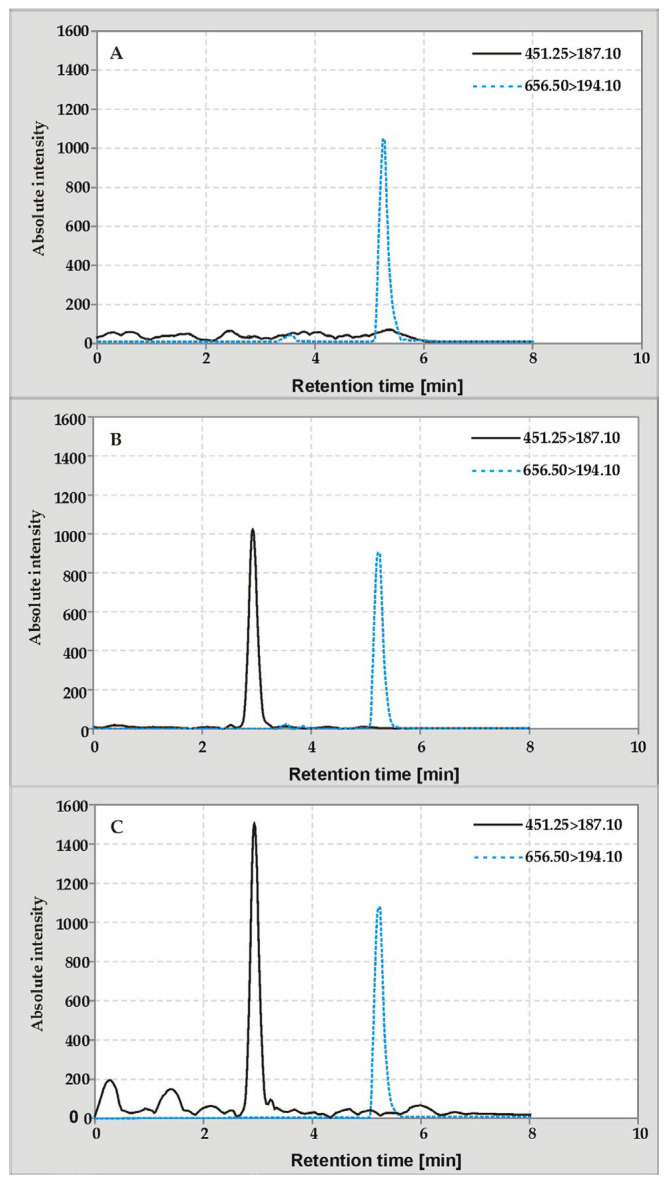
Multiple reaction monitoring chromatograms showing K1 and K2MK-7-D7 peaks marked with solid black and dashed blue lines, respectively, obtained for iceberg lettuce extracts using PLE (**A**,**B**) and UASE (**C**). Chromatogram A shows the extract obtained for a blank sample in which the analyte was destroyed by irradiation.

Currently, there is an increase in the use of LC-MS in the analysis of compounds present at low concentration levels in complex types of samples, mainly biological ones. These systems allow for a significant improvement in the signal-to-noise ratio as well as the specificity of the determination. Analytical software of modern instruments also allows for relatively easy modification of the existing procedures by enabling the addition of new MS/MS data packages for subsequent analytes. For this purpose, it is necessary to investigate and optimize the analyte fragmentation paths and collision energies for MRM transitions. This information collected for the K1 vitamer is presented in Section 3.4 and was used to modify the method described previously [16]. For the purpose of quantitative analysis, due to the high price of isotope-labeled standards, the deuterated K2MK-7 vitamer standard used in previous studies was applied. Exemplary MRM chromatograms of extracts obtained under optimized conditions of the PLE and UASE techniques for iceberg lettuce samples are shown in Figure 1. It is worth adding that, unlike the studies presented in [10,19,20,21,22], freeze-dried material was used in this study.

Figure 1 compares exemplary APCI(+)-MRM chromatograms obtained for the blank sample and the above-mentioned iceberg lettuce extracts obtained after the PLE and UASE procedures. Each sample was enriched with a deuterium derivative of vitamin K2MK-7-D7. Due to the lack of commercially available blank and control samples, a representative sample of iceberg lettuce was used in the experiments in which the endogenous vitamin K1 was destroyed by irradiation (chromatogram A). A detailed description of obtaining the blank sample together with a description of the validation experiments is presented in Section 3.4. 

The obtained chromatograms indicate the presence of the desired peaks, i.e., the analyte and the internal standard, which are well-separated from the peaks of the interfering substances. This allows us to state that the applied LC-MS/MS conditions are acceptable for both qualitative and quantitative analysis of vitamin K1 in plant extract samples. In order to assess the analytical utility of the modified method, its validation procedure was carried out. The results of the validation experiments (see also Supplementary Materials) are summarized in Table 1. They show that the method is characterized by good linearity, very low detection limits, and satisfactory inter- and intra-day precision for the quantitative analysis of phylloquinone in plant extracts. What is equally important is that the presented characteristics of the developed method do not differ from previously reported methodologies [16].

### 2.2. Extraction Optimization

Despite the significant development of extraction and separation techniques, the isolation of natural products from natural sources is still a difficult task. It requires an optimization process, which, despite the availability of useful mathematical tools in this regard, is carried out “on foot” optimizing one parameter after another in the so-called sequential extraction procedure. In this study, the central composite design (CCD) method was used to optimize extraction. 

As mentioned, in CCD, the distance from the center of the design space to the factor point depends on the properties desired for the design and on the number of factors involved. If the distance between the center of the design space and the factor point is ±1 unit for each variable, then the distance between the center of the design space and the star point is ±α, where |α| > 1. The CCD design applied in the study connected the vertices of a hypercube whose coordinates were given by the standard 2^n^ design. Three factors were used (temperature, pressure, and time); therefore, the factorial portion was equal to 2^3^ and the scaled value for α relative to 1 was 2^3/4^.

The layout of CCD with the decoded independent variables and the predicted values from the “main linear/square effects and the bidirectional interaction model” along with the experimental values of vitamin K1 yield obtained under given conditions of PLE from iceberg lettuce are summarized in Table 2. A mixture of *n*-hexane and ethyl acetate mixed in a volume ratio of 4:1 was used for extraction in the PLE experiment. The efficiency was demonstrated in studies on the optimization of the UASE procedure (see Supplementary Materials). 

The predicted values were determined using the response surface methodology and empirical relationships between vitamin K1 yield (Y) and independent variables, i.e., extraction temperature (X_1_), pressure (X_2_), and static extraction time (X_3_) based on the following equation in coded units:

(1) $$Y=26.3824+0.6453X_{1}-0.1422X_{2}+0.6519X_{3}-0.0034X_{1}^{2}+0.0072X_{2}^{2}+0.0072X_{3}^{2}+1.3441\cdot 10^{-5}X_{1}X_{2}-0.0082X_{1}X_{3}+0.0020\cdot 10^{-3}X_{2}X_{3}$$

To assess the quality of matching the mathematical model to the experimental data, analysis of variance (ANOVA), Fisher’s *F*-test at the 95% confidence level, as well as the coefficient of determination (R^2^) along with the adjusted determination coefficient (R_adj_^2^) were employed. For clarity, a low *p*-value indicates the statistical significance of the model. The closer the R^2^ value to 1, the better the correlation between the predicted and experimental data. All these results are summarized in Table 3.

The analysis of the data from the above table leads to the conclusion that among all the optimization parameters of the PLE process, it is the temperature that statistically significantly models the efficiency of vitamin K1 extraction. The calculated *F*-value for temperature, both in the linear and quadratic terms of the model, exceeds the tabular *F*-value (*F_tab_*) obtained after taking into account the appropriate degrees of freedom at the level of *F_tab_*_(9,7)_ = 3.67. This indicates the statistical significance of this parameter (*p* < 0.0001 for X_1_ and X_1_^2^, respectively). Similarly, statistically significant F values were obtained with bidirectional interactions of temperature and time (*p* < 0.0001 for X_1_ X_3_). The influence of the remaining parameters in the linear, quadratic, and two-way interactions is statistically insignificant.

As for the assessment of the quality of the model based on the value of the coefficient of determination (R^2^) and the adjusted coefficient of determination (R_adj_^2^), the value of R^2^ was 0.9614, which indicates that the mathematical model could provide 96.14% of the results. In turn, the analysis of the R_adj_^2^ value, which is a measure for testing the goodness of fit of the regression equation, shows that only 5.90% of the total variation was not explained by the model. Therefore, there is no statistical evidence to suggest that the developed model does not sufficiently explain the differentiation in the vitamin K1 yield from iceberg lettuce as a function of variable extraction parameters, i.e., temperature, pressure, and static extraction time. Thus, it should be assumed that the statistical analysis reveals the correctness of the developed model to predict optimal conditions for the extraction of vitamin K1.

### 2.3. Variables Affecting PLE

**Figure 2:**
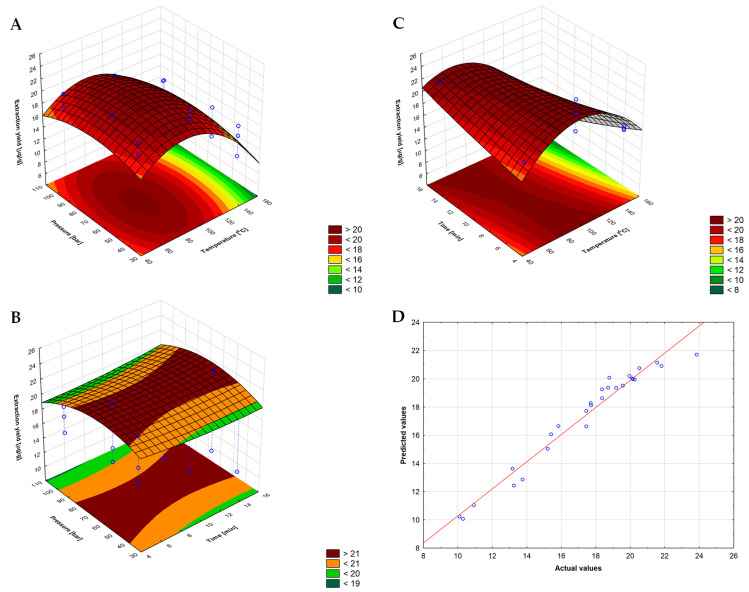
The 3D surface plots for the vitamin K1 yield from iceberg lettuce as a function of temperature and pressure (**A**), pressure and time (**B**), temperature and time (**C**) using PLE as well as plot of predicted vs. measured (actual) K1 extraction efficiency values (**D**).

The 3D surface plots shown in Figure 2 allow for a more understandable comparison of the interaction effects of independent and dependent variables. These graphs were obtained by changing the values of two independent variables, keeping the third variable constant and predicting the efficiency of vitamin K1 extraction from iceberg lettuce. Thus, the influence of temperature and pressure during the 10-min extraction time is shown in Figure 2A; the effect of extraction time and pressure at 85 °C is presented in Figure 2B; and the influence of time and temperature on the extraction efficiency at a pressure of 66 bar is shown in Figure 2C. Figure 2D shows a graph of the values predicted by the developed model as a function of the actual vitamin K1 extraction efficiency.

The analysis of the relationships presented in Figure 2 confirms that temperature and time are parameters that significantly change the efficiency of vitamin K1 extraction. With longer exposure of the sample to temperatures above approximately 110 °C, the recovery of vitamin K1 decreases significantly as a result of thermal degradation of the analyte [19,23]. In turn, shortening the exposure time at temperatures below approximately 80 °C similarly leads to lower extraction efficiency, but this time due to a poorer rate of analyte mass transfer between phases. Therefore, the observed trend of changes (resultant effect) is a combination of two competing processes: an increase in the efficiency of isolation of the analyte from the plant matrix and the degradation of the analyte released from the matrix. This trend is also visible in Figure 2A and to a lesser extent in Figure 2B.

As for the other PLE parameters, their impact on the extraction efficiency is smaller. Nevertheless, contrary to the data presented in [19], it is worthwhile to slightly extend the static extraction time from 5 min to 8 min in order to effectively isolate vitamin K1 in a single extraction cycle, extending the contact of the plant matrix with the extractant at an elevated extraction temperature. Similarly, unlike the data presented in the previously quoted paper, the effective extraction of vitamin K1 is favored by lower process pressure, with the optimal pressure being 66 bar. Reducing the extraction to a single extraction cycle conducted at lower pressure will undoubtedly increase the profitability of vitamin K1 extraction using the PLE technique at an industrial scale.

### 2.4. Model Verification

The usefulness of the model for predicting the optimal efficiency of vitamin K1 extraction was checked for iceberg lettuce by comparing the yield predicted by the model with the experimental value determined under adapted extraction conditions, i.e., 85 °C, 66 bar for 8 min. The vitamin K1 yield predicted by the model under the given extraction conditions was 21.28 µg/g. The experimental value was 19.94 ± 1.39 µg/g. The ANOVA analysis did not confirm statistically significant differences between the values obtained experimentally and those predicted by the model (*F_cal_* = 0.29, *F_tab_* = 18.51). It can therefore be assumed that the designed model explains the actual process of vitamin K1 extraction.

### 2.5. Application of Mathematically Designed Optimal PLE Conditions to Extract Vitamin K1 from Various Vegetables and Fruits 

As mentioned, there is little literature data on the content of vitamin K1 determined in a larger group of food products. In addition, due to the use of different methods of extracting vitamin K1 from plant matrices and different analytical techniques, the sporadic literature data obtained in different research groups are difficult to compare [10,20,21,22,27]. It should also be remembered that the content of compounds synthesized by plants is differentiated by the seasonal variability of insolation or humidity. The content of compounds is also modified by transport and storage conditions. Taking the above into account and remembering the health-promoting role of vitamin K1, in the next stage of the research, it was decided to use mathematically designed optimal PLE conditions to extract vitamin K1 from various commonly consumed vegetables and fruits available in one of the nationwide chain stores. In order to compare and control the effectiveness of extraction using the PLE technique, another extraction technique was used in the study, also included in the assisted extraction techniques, namely the UASE technique [26,28].

**Figure 3:**
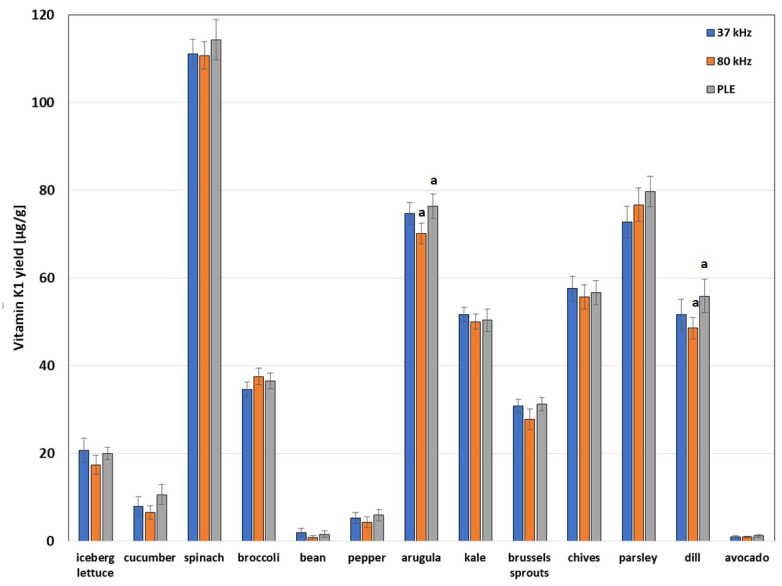
The content of vitamin K1 in plants revealed by the PLE technique under the conditions provided by the mathematical model and the developed and optimized UASE technique at two frequencies, i.e., 37 kHz and 80 kHz. The letters in the figure indicate data for which the differences in K1 content are statistically significant (*F_cal_* > *F_tab_*, *p* > 0.05).

The results of this series of studies are presented in Figure 3. Their analysis shows that the content of vitamin K1 in the plants tested is very diverse. In general, the highest content of this analyte was found in spinach, which is consistent with the literature data [27,29]. Nevertheless, high vitamin K1 content is also characteristic of arugula, parsley, chives, dill, and kale, respectively. The smallest amounts were found in broad beans and avocado. In the case of the vast majority of vegetables and fruits tested, the differences in the results obtained using the UASE technique at two ultrasound frequencies and the PLE technique are statistically insignificant. This confirms the accuracy of the vitamin K1 determination in the plants tested. Only in the case of arugula and dill is the efficiency of PLE statistically significantly higher than the efficiency of vitamin K1 extraction using the UASE technique at a frequency of 80 kHz. It is worth recalling here that the PLE extraction time is more than twice shorter than the UASE time. Vitamin K1 in the plant matrix may interact with other matrix components and it is likely that the higher temperature of the PLE process favors the release of this analyte from stronger interactions, which may explain the lower efficiency of the UASE extraction carried out at lower temperatures; however, confirmation of this assumption requires another series of experiments. The literature data showing a higher yield of vitamin K1 from spinach subjected to prior heat treatment, in comparison with the yield of the analyte from raw spinach, may indirectly confirm the validity of the above assumption [27]. A similar situation occurs in the case of the isolation of carotenoids from plants [29,30]. However, it should be remembered that the amount of the compound extracted from the plant material is the result of two opposing processes occurring in parallel, i.e., extraction and degradation of the analyte under the influence of temperature.

### 2.6. Effect of Water on the Efficiency of Vitamin K1 Extraction from Plant Matrices

Vitamin K1 is a hydrophobic compound, therefore, hydrophobic extractants are commonly used for its extraction, according to the rule “like dissolves like” [19,20,21,22]. A fairly common practice in the nutritional studies of natural products is to use unprepared material, possibly subjected only to grinding. In another approach, the plant material is dried and the residual water after drying is generally not determined. Bearing in mind that variable water content can differentiate the effectiveness of vitamin K1 extraction, in the next series of studies, it was decided to check whether this is indeed the case.

**Figure 4:**
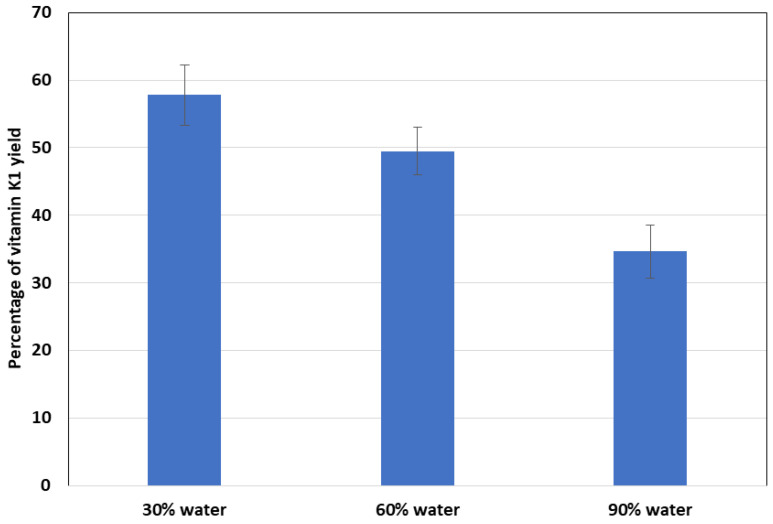
Change in the yield of vitamin K1 from iceberg lettuce after introducing increasingly more water into the extraction system.

The results of this series of studies are summarized in Figure 4 as a percentage of the vitamin K1 extraction efficiency from iceberg lettuce after introducing increasing amounts of water to the extraction system. The water content was expressed as a percentage of the mass of the freeze-dried iceberg lettuce sample, i.e., 30%, 60%, and 90% water, respectively. The yield obtained for the freeze-dried material was taken as a reference point.

The analysis of the presented dependencies leads to the conclusion that the higher the water content in the extracted sample, the lower the analyte extraction yield. Water present in the extracted sample most likely reduces the contact surface between the phases and hinders the release of the analyte into the volume of the nonpolar extractant. Thus, the lack of drying of the plant material leads to erroneous results regarding the vitamin K1 content of the plant material. This fact is another reason for the need to standardize the analytical methods used in the assessment of the properties of food products.

## 3. Materials and Methods

### 3.1. Plant Materials and Chemicals

All the plants that were the subject of this study were purchased at a local store (Lublin, Poland). Representative amounts of each vegetable, i.e., iceberg lettuce (*Lactuca sativa* var. *capitata*), spinach (*Spinacia oleracea*), broccoli (*Brassica oleracea* var. *italica*), cucumber (*Cucumis sativus*), bell pepper (*Capsicum annuum*), bean (*Vicia faba*), arugula (*Eruca vesicaria* ssp. *sativa*), brussels sprouts (*Brassica oleracea* var. *gemmifera*), kale (*Brassica oleracea*), chives (*Allium schoenoprasum*), parsley (*Petroselinum crispum*), dill (*Anethum graveolens* L.), and avocado (*Persea americana* Mill.) were pre-frozen in a freezer and then freeze-dried using a Labconco Centrivap Cold Trap (−84 °C). During freeze-drying, the moisture level of the plants was checked every 50 min and the process was continued until the change in mass loss between subsequent measurements was less than 0.1 g. Dried samples were ground with a Braun cutting mill to a particle size of 0.2–0.4 mm and passed through a sieve. Accurately weighed portions of the samples were used for extraction.

Methanol (MeOH) and acetonitrile (ACN), both LC/MS grade, were purchased from Merck (Darmstadt, Germany). Ethyl acetate and *n*-hexane (both with analytical purity grade) were purchased from the Polish Chemicals Plant POCh (Gliwice, Poland). The 99% formic acid of LC-MS purity was acquired from VWR Chemicals (Gdańsk, Poland). The standards of vitamin K1 and deuterium-labeled K2MK-7, used as an internal standard (K2MK-7-D7), were obtained from Sigma-Aldrich (St. Louis, MO, USA). Sand was obtained as a gift from glassworks (fraction 0.4–0.6 mm). Water was purified using the Milli-Q system (Millipore Sigma, Bedford, MA, USA). 

All samples used in this study were kept in amber non-transparent vials unless stated otherwise. 

### 3.2. Pressurized Liquid Extraction

PLE was performed with a Dionex instrument (Dionex Corp., Sunnyvale, CA, USA). Each plant material (0.1 g) was mixed with neutral glass to reduce the solvent volume used for extraction, placed into a 22 mL stainless steel extraction cell, and subjected to the extraction procedure. 

During the optimization experiments, the influence of extraction temperature (50, 100, and 150 °C) on the extraction efficiency of vitamer K1 was studied at constant extraction time (10 min) and pressure (60 bar); the influence of the extraction pressure (40, 60, and 100 bar) was studied at constant temperature 100 °C and extraction time (10 min); and, finally, the influence of extraction time (5, 10, and 15 min) was explored at constant temperature 100 °C and pressure (60 bar). 

All PLE extractions were performed using the *n*-hexane/ethyl acetate mixture (4:1 *v*/*v*) as extractant. The preset default PLE procedure was as follows: solvent flush volume equal to 100% of the extraction cell volume; purge time 60 s using pressurized nitrogen (10 bar). The extracts were collected in 60 mL glass vials with Teflon-coated rubber caps. The system was washed with extraction solvent between runs. The obtained extracts (volumes of ca. 35 mL) were transferred into volumetric flasks (50 mL) and filled with extractant to their volume. Prior to chromatographic analysis, 1 mL of the extract was evaporated to dryness under a stream of nitrogen and then reconstituted in the same volume of methanol with 0.1% formic acid.

### 3.3. Ultrasound-Assisted Solvent Extraction

Ultrasound-assisted solvent extraction (UASE) was carried out in an Elmasonic P ultrasonic bath (Elma, Singen, Germany) under controlled and optimized extraction conditions—extraction time: 20 min, frequency: 37 and 80 kHz, temperature: 50 °C, power: 100%—using an extraction mixture of *n*-hexane/ethyl acetate (4:1, *v*/*v*) [26]. Optimization experiments were carried out using iceberg lettuce. During the experiments, the influence of the type of solvent (methanol, acetone, *n*-hexane/ethyl acetate (4/1, *v*/*v*), *n*-hexane), extraction time (5, 10, 15, and 20 min), and the ultrasonic generator power on the yield of vitamin K1 was checked. For extraction, portions of freeze-dried plant samples (0.1 g) were placed in tightly closed centrifuge tubes with 15 mL of extraction mixture. Centrifugation (10 min, 2700 rpm) was used to separate the extract obtained from the plant material. Then, the extracts were poured into 50 mL volumetric flasks that were filled to volume with the extraction mixture. Before analysis, 1 mL of the extract was evaporated to dryness under a stream of nitrogen and then reconstituted in the same volume of methanol with 0.1% formic acid.

### 3.4. LC-MS Analysis, Validation, and Statistical Analysis

The chromatographic analyses were performed using a previously described procedure [16], which was modified and validated for the quantitative determination of vitamer K1. Due to the high cost of deuterated standards, the determination was carried out against the deuterium derivative of vitamin K2MK-7 (K2MK-7-D7) used in the previous study as an internal standard (IS). 

The measurements were collected on a Shimadzu NEXERA X2 LC system (Shimadzu, Kyoto, Japan) equipped with a double binary pump (LC-30AD), a system controller (CBM-20A), an automatic solvent degasser (DGU-20A5R), and an autosampler (SIL-30AC) maintained at 20 °C. Separations were performed using a Kinetex C18 column (50 × 2.1 mm, 2.6 μm, Phenomenex, Inc., Torrance, CA, USA) applying gradient elution with the mobile phase flow at 0.6 mL/min. The mobile phase A was the solution of 0.1% formic acid in water; the mobile phase B was 0.1% formic acid in MeOH. The gradient program started with an increase from 90% B to 100% B in 4 min, then 100% B was maintained for another 4 min, and finally, the column was equilibrated with the initial composition of the mobile phase, i.e., 90% B for 10 min. The total run time was 18 min with the first 8 min monitored. The column temperature was maintained at 40 °C and controlled with the column oven (CTO-20AC). The injected sample volume was reduced to 2 µL. 

Detection was performed with an LCMS-8050 triple-quadrupole mass spectrometer (Shimadzu, Kyoto, Japan) equipped with an atmospheric pressure chemical ionization (APCI) source operating in the positive ion mode under the following conditions: APCI temperature 350 °C, the desolvation line temperature 200 °C, the heating block temperature 200 °C, the nebulizer gas flow 3 L/min, and the drying gas flow 5 L/min. Nitrogen was used as the collision gas.

Identification and quantification were performed in the multiple reaction monitoring (MRM) mode after examination of the analyte fragmentation spectrum and IS according to the criteria for identity confirmation specified in Commission Decision 2002/657/EC. According to them, MRM measurements for the analyte were performed for two transitions: *m*/*z*^+^ 451.3 → 105.1 (qualifier transition, S2) and 451.3 → 187.1 (quantifier transition, S1), and one transition *m*/*z*^+^ 656.0 → 194.1 for K2MK-7-D7 using the collision energy (CE) values at 45 eV, 30 eV, and 35 eV, respectively. To determine the optimal MS/MS operating conditions, the analyte standard and IS, each at a concentration of 100 ng/mL, were separately injected into the LC-MS/MS system and the mass transitions and corresponding collision energies were optimized for each compound.

The method was validated for linearity, limit of detection (LOD), limit of quantification (LOQ), and intraday and interday precision and accuracy measurements [31]. Validation studies were carried out on the extract obtained from iceberg lettuce. For this purpose, lettuce portions were subjected to PLE extraction carried out under default conditions (10 min, 100 °C, 60 bar) using a mixture of *n*-hexane/ethyl acetate (4:1 *v*/*v*). Then, individual extracts were exposed to UV irradiation to photodegrade the endogenous vitamin K1 and afterward, after checking the absence of the analyte, the extracts were combined to obtain a representative portion of the blank sample.

To establish a calibration curve, the aliquots of the blank sample were spiked with the sequentially increased amounts of the phylloquinone standard and the same amount of IS (10 ng/mL). Seven calibration samples were examined, including the blank sample, the zero sample (the blank sample with added IS), and five non-zero samples in the range of 1.0 ng/mL to 100 ng/mL of K1. To assess the linearity of the method, three replicated analytical procedures were applied independently for each examined concentration level. A matrix-matching calibration curve was constructed by plotting the peak area ratio of K1 to IS against the known K1 concentration. The slope, intercept, and coefficient of determination (R^2^) were determined by the least squares linear regression model. The quality of calibration was evaluated by the back-calculation of K1 concentrations in the calibration solutions. The calibration solution with the lowest analyte concentration was used to estimate LOD and LOQ. The LOD and LOQ were considered as signal-to-noise ratios of 3 and 10, respectively. 

The intra- and inter-day precisions and accuracy were assessed by statistical analysis of the quantitative results obtained on the same day and on three different days for five independent control samples prepared at concentrations of 25 ng/mL and 75 ng/mL. The precision was estimated using the one-way analysis of variance (ANOVA) test, and it was expressed as the coefficient of variation (CV, %). The accuracy evaluation was made comparing the mean value of the obtained results to the nominal concentration level of the analyte in the control sample using the Student’s *t*-test and it was expressed as BIAS (in %). 

Recovery was assessed using iceberg lettuce samples extracted with PLE under optimal conditions that were fortified with the analyte and IS at concentrations of 25 ng/mL and 75 ng/mL immediately prior to extraction. It was calculated as the percentage of the analyte response compared to the response from a solution containing the analyte at a concentration corresponding to 100% recovery. Analysis of variance (ANOVA) was performed to determine whether there was a significant difference between the recovery percentages at each analyte concentration level.

Matrix effect and recovery were determined using the area of the K1 and IS peaks in two differently prepared sets of samples, each at concentrations of 25 ng/mL and 75 ng/mL [32]. The first set was composed of standard solutions prepared in solvents used as the extractant mixture instead of a blank sample. The second was prepared in the aliquots of the blank sample fortified with the vitamin and IS. ANOVA was performed to determine if there was a significant difference between the peaks at a given analyte concentration level.

The specificity of the method was assessed by analyzing a blank sample for the absence of peaks at the retention time of the analyte and internal standard.

### 3.5. Experimental Design and Statistical Analysis

A statistical experiment based on central composite design and Statistica10 software (StaSoft, Inc., Tulsa, OK, USA) was used to determine the effect of three independent variables and the optimum conditions of vitamer K1 extraction. The effects of the variables temperature, pressure, and time of the extraction process coded as *X*_1_, *X*_2,_ and *X*_3_ were investigated at five levels (−1.68179, −1, 0, 1, 1.68179). The dependent variable was *Y*, i.e., as large as possible for the vitamin K1 yield from 1 g of iceberg lettuce. The validity of the model was evaluated using ANOVA analysis, the determination coefficient (*R*^2^), and the adjusted determination coefficient (*R_adj_*^2^). In addition, the optimal condition was verified by conducting experiments under these conditions.

All experimental data are presented as the mean values of three independent measurements unless otherwise stated, ±standard deviation (SD). *p* ≤ 0.05 was used to declare the statistically significant terms with a 95% confidence level.

## 4. Conclusions

Recently, awareness of the various health benefits of vitamin K consumption has attracted the attention of scientists in many fields, including food chemists, to discover more effective methods of its extraction and more accurate and precise methods of its analysis in complex matrices. In these efforts, attention is also focused on using methods that are not only more efficient but also more economical and ecological. An important argument in favor of the possibility of scaling procedures from a laboratory to an industrial scale is the automation of the process. Hence, the so-called assisted extraction techniques are of great interest. In terms of the target analytical technique, there is currently a shift from less selective high-performance liquid chromatography (HPLC) methods to very sensitive and more selective LC-MS methodologies, which is associated with a lower cost of the latter devices.

This article proposes a procedure for the extraction and analysis of vitamin K1 using PLE and LC-APCI-MS. It has been shown that the PLE technique can be optimized with a mathematical model—accelerating and reducing the costs of the extraction process—which, together with process automation, bodes well for industrial applications. The optimized process was used to extract vitamin K1 from iceberg lettuce, cucumber, spinach, broccoli, bean, bell pepper, arugula, kale, brussels sprouts, chives, parsley, dill, and avocado, showing very different contents of the test compound ranging from 1.22 µg/g to 114.30 µg/g dry weight for avocado and spinach, respectively. By showing the influence of water in the extracted material on the variable efficiency of K1 isolation from the same material, attention was drawn to the need to standardize the analytical methods used in assessing the quality of food products. Moreover, it was hypothesized that thermal processing of vegetables promotes the release of larger amounts of this valuable health compound. This hypothesis requires proof but is intriguing nonetheless.

There is not much information in the literature on the content of vitamin K in various food groups, including fruits and vegetables. Most of the available information is limited to single selected and characteristic regional products. This study complements this lack of knowledge by showing in a broader perspective the variable content of vitamin K1, which is valuable for health, in commonly consumed fruits and vegetables.

## Figures and Tables

**Table 1:** Results of validation for the LC-MS/MS method.

Validation Parameter	Phylloquinone Analysis
Linearity (R^2^)	0.9968
Intraday precision (% CV)	3.95–5.55
Interday precision (% CV)	5.08–5.84
Intraday accuracy (% BIAS)	−3.88–10.72
Interday accuracy (% BIAS)	−4.36–8.15
LOD (ng/mL)	0.024
LOQ (ng/mL)	0.072
Recovery	Recovery percentages estimated using optimal extraction conditions were more than 90%
Matrix effect	No significant differences were found between the signal ratios of analyte to IS at both analyte concentration levels; therefore, the presented method is not subject to matrix influence.
Selectivity	The absence of peaks in the retention time of the analyte and IS and/or significant interferences in the chromatogram confirms the selectivity of the presented method.

**Table 2:** Central composite design arrangement as well as experimental and predicted values for vitamin K1 yield from iceberg lettuce.

Run	Independent Variables			Vitamin K1 Yield (Y) [µg/g]	
	Temperature	Pressure	Time	Experimental	Predicted
	X1	X2	X3		
1	50	40	5	17.45	17.72
2	50	40	10	18.37	18.62
3	50	40	15	20.26	19.95
4	50	60	5	17.70	18.27
5	50	60	10	18.72	19.38
6	50	60	15	21.81	20.90
7	50	100	5	17.45	16.62
8	50	100	10	17.73	18.12
9	50	100	15	20.11	20.03
10	100	40	5	21.56	21.13
11	100	40	10	20.15	19.98
12	100	40	15	18.37	19.25
13	100	60	5	23.85	21.70
14	100	60	10	20.53	20.74
15	100	60	15	19.95	20.21
16	100	100	5	18.79	20.07
17	100	100	10	19.57	19.51
18	100	100	15	19.19	19.37
19	150	40	5	15.41	16.06
20	150	40	10	13.76	12.85
21	150	40	15	10.31	10.07
22	150	60	5	15.83	16.64
23	150	60	10	13.18	13.63
24	150	60	15	10.93	11.04
25	150	100	5	15.21	15.04
26	150	100	10	13.26	12.42
27	150	100	15	10.10	10.22

**Table 3:** Analysis of variance for the second-order polynomial model estimated for the vitamin K1 yield obtained from iceberg lettuce using PLE.

Variation Source	The Vitamin K1 Yield				
	Sum of Square (SS)	Degree of Freedom (df)	Mean Square (MS)	*F*-Value	*p*-Value
X_1_	145.22	1	145.22	190.15	<0.0001
X_2_	0.99	1	0.99	1.29	0.2716 *
X_3_	7.41	1	7.41	9.71	0.0063
X_1_^2^	107.50	1	107.50	141.34	<0.0001
X_2_^2^	4.91	1	4.91	6.42	0.0214
X_3_^2^	0.26	1	0.26	0.34	0.5651 *
X_1_ X_2_	0.01	1	0.01	0.01	0.9361 *
X_1_ X_3_	50.71	1	50.71	66.41	<0.0001
X_2_ X_3_	1.07	1	1.07	1.40	0.2524 *
Residual	12.98	17	44.49		
Cor total	336.32	26			
R^2^	0.9614				
R_adj_^2^	0.9410				

*—not significant.

## Data Availability

The original contributions presented in this study are included in the article; further inquiries can be directed to the corresponding author.

## Supplementary Materials

The following supporting information can be downloaded at: https://www.mdpi.com/article/10.3390/molecules29184420/s1, Figure S1: Influence of the extractant type on the vitamin K1 extraction yield from iceberg lettuce in UASE; Table S1: ANOVA results; Table S2: Method of calculating the F value together with statistical analysis; Figure S2: Effect of UASE conditions on K1 extraction efficiency in (μg/g) from iceberg lettuce; Table S3: Summary of the statistical analysis of the effect of UASE extraction conditions on K1 yield; Table S4: Comparison of the concentrations values of calibration solutions with the concentrations calculated from the equation of calibration curve, along with statistical analysis; Figure S3: Calibration curve graph with confidence intervals; Figure S4: Residue distribution graph; Table S5: Summary of the statistical analysis of calibration; Table S6: Results of the lack of fit test; Table S7: Intraday precision (% CV) and accuracy (BIAS); Table S8: Interday precision (% CV) and accuracy (BIAS); Table S9: ANOVA results for the interday precision study at concentration level 25 ng/mL; Table S10: ANOVA results for the intraday precision study at concentration level 25 ng/mL; Table S11: ANOVA results for interday precision study at concentration level 75 ng/mL; Table S12: ANOVA results for intraday precision study at concentration level 75 ng/mL; Table S13: Summary of the accuracy study at *p* < 0.05; Table S14: Recovery.
