# Supplementary material for: Quantitative Study of Vitamin K in Plants by Pressurized Liquid Extraction and LC-MS/MS
Source: Molecules. 2024 Sep 17;29(18):4420. doi: 10.3390/molecules29184420 (PMC11434174; doi:10.3390/molecules29184420)
Supplement: Supplementary file 1 [file molecules-29-04420-s001.zip › molecules-3173512-supplementary.pdf]

## Supplementary Materials

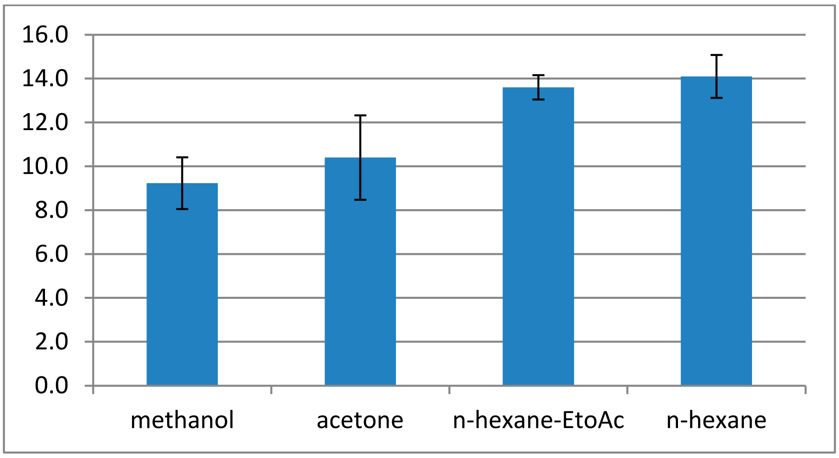


**Figure S1.** Influence of the extractant type on the vitamin K1 extraction yield from iceberg lettuce in UASE.

**Table S1.** ANOVA results

| **Summary** | | | | | | |
|---|---|---|---|---|---|---|
| *Groups* | *Count* | *Sum* | *Average* | *Variance* | | |
| methanol | 3 | 27.7 | 9.233333 | 2.093333 | | |
| acetone | 3 | 31.2 | 10.4 | 5.5696 | | |
| n-hexane-EtoAc | 3 | 40.8 | 13.6 | 0.4624 | | |
| n-hexane | 3 | 42.3 | 14.1 | 1.44 | | |
| | | | | | | |
| **ANOVA** | | | | | | |
| *Source of variation* | *SS* | *df* | *MS* | *F* | *p-value* | *p-value* |
| Between Groups | 51.22 | 3 | 17.07333 | 7.139671 | 0.011899 | 4.066181 |
| Within Groups | 19.13067 | 8 | 2.391333 | | | |
| Total | 70.35067 | 11 | | | | |
| | | | | | | |
| **Summary** | | | | | | |
| *Groups* | *Count* | *Sum* | *Average* | *Variance* | | |
| n-hexane-EtoAc | 3 | 40.8 | 13.6 | 0.4624 | | |
| n-hexane | 3 | 42.3 | 14.1 | 1.44 | | |
| | | | | | | |
| **ANOVA** | | | | | | |
| *Source of variation* | *SS* | *df* | *MS* | *F* | *p-value* | *p-value* |
| Between Groups | 0.375 | 1 | 0.375 | 0.394239 | 0.564144 | 7.708647 |
| Within Groups | 3.8048 | 4 | 0.9512 | | | |
| | 4.1798 | 5 | | | | |

**Table S2.** Method of calculating the F value together with statistical analysis

| | | *Predicted* | *Experimental* |
|---|---|---|---|
| run 4 | 18.27 | 22.35803 | 19.94 |
| run 5 | 19.38 | 20.20197 | 21.33 |
| run 6 | 20.9 | 21.28 | 18.55 |
| mean | 19.51667 | 21.28 | 19.94 |
| SD | 1.078033 | 1.078033 | 1.39 |

| **Summary** | | | | | | |
|---|---|---|---|---|---|---|
| *Groups* | *Count* | *Sum* | *Average* | *Variance* | | |
| 22,35803 | 2 | 41.48197 | 20.74098 | 0.581078 | | |
| 19,94 | 2 | 39.88 | 19.94 | 3.8642 | | |
| | | | | | | |
| **ANOVA** | | | | | | |
| *Source of variation* | *SS* | *df* | *MS* | *F* | *p-value* | *F crit* |
| Between Groups | 0.641574 | 1 | 0.641574 | 0.288654 | 0.644861 | 18.51282 |
| Within Groups | 4.445278 | 2 | 2.222639 | | | |
| Total | 5.086852 | 3 | | | | |

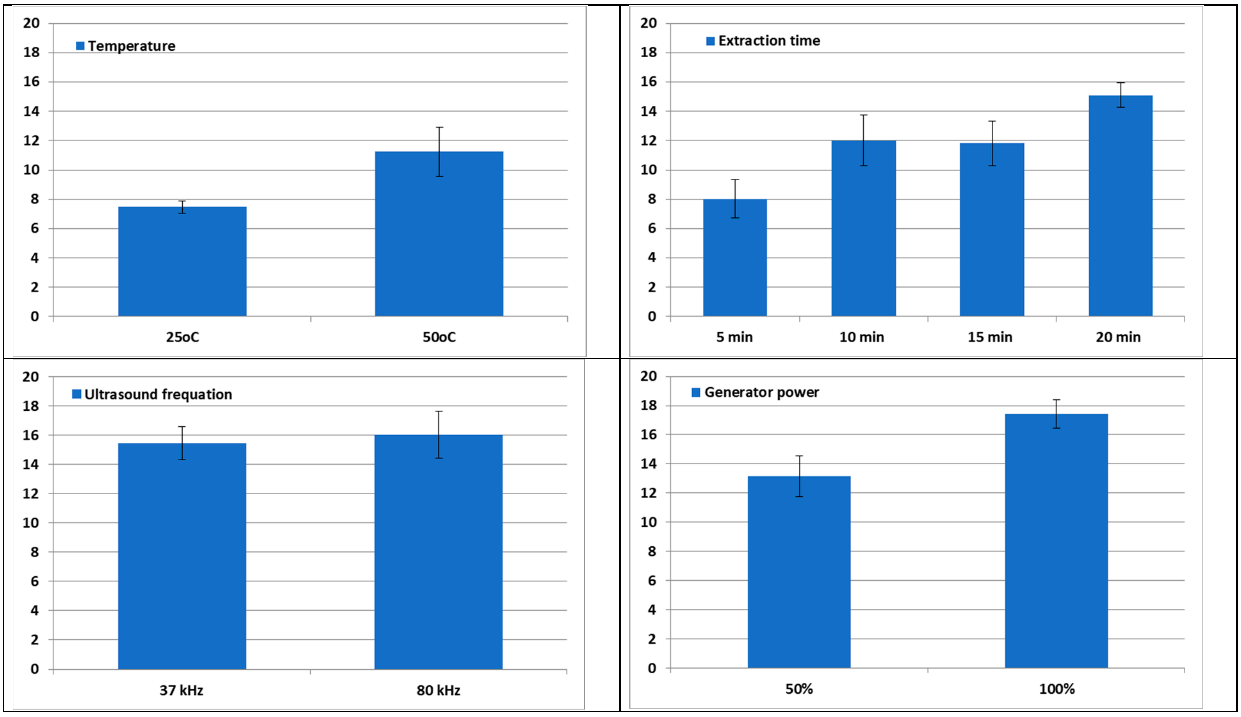

**Figure S2** Effect of UASE conditions on K1 extraction efficiency in (µg/g) from iceberg lettuce.

**Table S3** Summary of the statistical analysis of the effect of UASE extraction conditions on K1 yield

**Effect of temperature**

Summary

| *Groups* | *Count* | *Sum* | *Average* | *Variance* |
|---|---|---|---|---|
| 25°C | 3 | 22.42 | 7.473333 | 0.173733 |
| 50 °C | 3 | 33.72 | 11.24 | 2.8308 |

ANOVA

| *Source of variation* | *SS* | *df* | *MS* | *F* | *p-value* | *F crit* |
|---|---|---|---|---|---|---|
| Between Groups | 21.28167 | 1 | 21.28167 | 14.16637 | 0.01971 | 7.708647 |
| Within Groups | 6.009067 | 4 | 1.502267 | | | |
| Total | 27.29073 | 5 | | | | |

**Effect of time**

Summary

| *Groups* | *Count* | *Sum* | *Average* | *Variance* |
|---|---|---|---|---|
| 5 min | 3 | 24.1 | 8.033333 | 1.730533 |
| 10 min | 3 | 36.04 | 12.01333 | 2.966933 |
| 15 min | 3 | 35.46 | 11.82 | 2.2828 |
| 20 min | 3 | 45.3 | 15.1 | 0.6772 |

ANOVA

| *Source of variation* | *SS* | *df* | *MS* | *F* | *p-value* | *F crit* |
|---|---|---|---|---|---|---|
| Between Groups | 75.33023 | 3 | 25.11008 | 13.11665 | 0.001865 | 4.066181 |
| Within Groups | 15.31493 | 8 | 1.914367 | | | |
| Total | 90.64517 | 11 | | | | |

**Effect of frequation**

Summary

| *Groups* | *Count* | *Sum* | *Average* | *Variance* |
|---|---|---|---|---|
| 37 kHz | 3 | 46.29 | 15.43 | 1.2817 |
| 80 kHz | 3 | 48.06 | 16.02 | 2.5428 |

ANOVA

| *Source of variation* | *SS* | *df* | *MS* | *F* | *p-value* | *F crit* |
|---|---|---|---|---|---|---|
| Between Groups | 0.52215 | 1 | 0.52215 | 0.273055 | 0.628895 | 7.708647 |
| Within Groups | 7.649 | 4 | 1.91225 | | | |
| Total | 8.17115 | 5 | | | | |

**Effect of generator power**

Summary

| *Groups* | *Count* | *Sum* | *Average* | *Variance* |
|---|---|---|---|---|
| 50% | 3 | 39.44 | 13.14666667 | 1.978133333 |
| 100% | 3 | 52.26 | 17.42 | 0.9172 |

ANOVA

| *Source of variation* | *SS* | *df* | *MS* | *F* | *p-value* | *F crit* |
|---|---|---|---|---|---|---|

| | | | | | | |
|---|---|---|---|---|---|---|
| Between Groups | 27.39207 | 1 | 27.39207 | 18.92153 | 0.012158 | 7.708647 |
| Within Groups | 5.790667 | 4 | 1.447667 | | | |
| | | | | | | |
| Total | 33.18273 | 5 | | | | |

**Table S4** Comparison of the concentrations values of calibration solutions with the concentrations calculated from the equation of calibration curve, along with statistical analysis

| | Nominal concentration [mg/mL] | Calculated concentration [mg/mL] | CV | Bias |
|---|---|---|---|---|
| KK_blank_001.lcd | 0 | 0.475 | 6.27796 | #DZIEL/0! |
| KK_blank_002.lcd | | | | |
| KK_blank_003.lcd | | | | |
| KK_1-1_004.lcd | 1 | 1.227 | 0.096445 | 22.7198 |
| KK_1-2_005.lcd | | | | |
| KK_1-3_006.lcd | | | | |
| KK_2-1_007.lcd | 25 | 22.949 | 0.102928 | -8.20359 |
| KK_2-2_008.lcd | | | | |
| KK_2-3_009.lcd | | | | |
| KK_3-1_010.lcd | 50 | 51.121 | 0.035082 | 2.242462 |
| KK_3-2_011.lcd | | | | |
| KK_3-3_012.lcd | | | | |
| KK_4-1_013.lcd | 75 | 71.055 | 0.043262 | -5.26011 |
| KK_4-2_014.lcd | | | | |
| KK_4-3_053.lcd | | | | |
| KK_5-1_016.lcd | 100 | 102.411 | 0.043732 | 2.411386 |
| KK_5-2_017.lcd | | | | |
| KK_5-3_018.lcd | | | | |

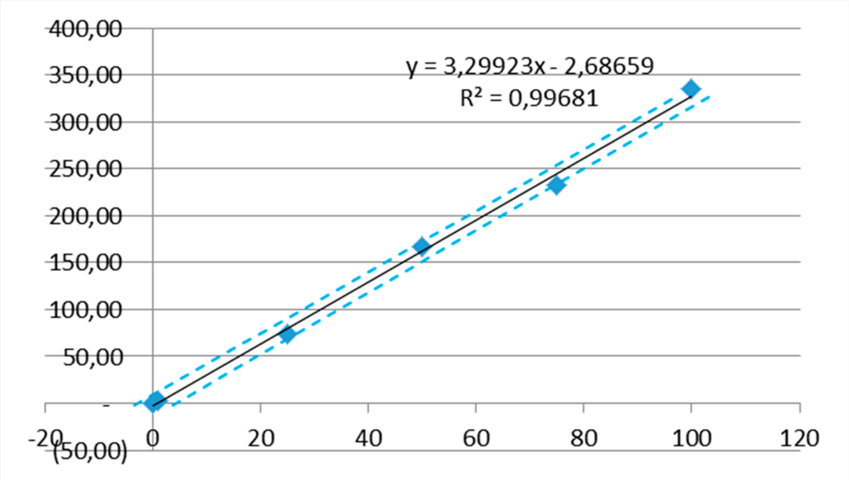


**Figure S3** Calibration curve graph with confidence intervals

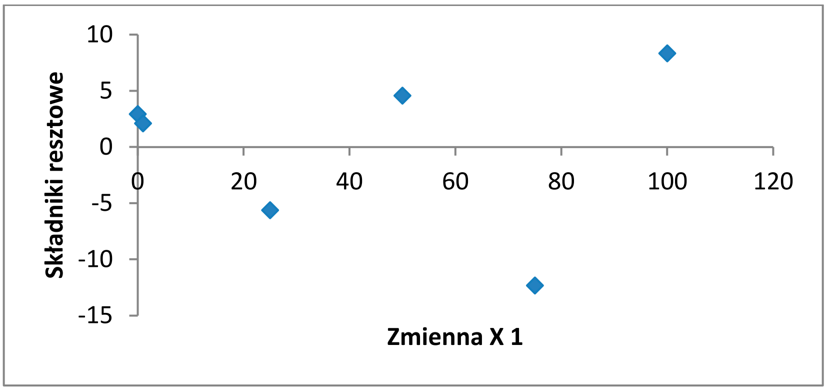


**Figure S4** Residue distribution graph

**Table S5** Summary of the statistical analysis of calibration

Summary output

| *Regression statistics* | |
|---|---|
| Multiple R | 0.998405248 |
| R Square | 0.99681304 |
| Adjusted R Square | 0.9960163 |
| Standard Error | 8.472525616 |
| Observations | 6 |

Variance analysis

| | *df* | *SS* | *MS* | *F* | *Significance F* |
|---|---|---|---|---|---|
| Regression | 1 | 89809.62 | 89809.62 | 1251.115 | 3.81282E-06 |
| Residual | 4 | 287.1348 | 71.78369 | | |
| Total | 5 | 90096.76 | | | |

| | *Coefficients* | *Standard Error* | *t Stat* | *p-value* | *Lower 95%* | *Upper 95%* | *Lower 95.0%* | *Upper 95.0%* |
|---|---|---|---|---|---|---|---|---|
| Intercept | -2.686586882 | 5.214355 | -0.51523 | 0.633559 | -17.1639566 | 11.79078286 | -17.163957 | 11.79078 |
| X Variable 1 | 3.299229496 | 0.093275 | 35.3711 | 3.81E-06 | 3.040257339 | 3.558201653 | 3.04025734 | 3.558202 |

**Table S6** Results of the lack of fit test

| Concentration (mg/ml) | Signal 1 | Signal 2 | Signal 3 | Mean signal | Signal_calc (au) | (mean-calculated)^2 | MSS (LoF) | | (Signal1(i)-mean_signal(i))^2 | (Signal2(i)-mean_signal(i))^2 | (Signal3(i)-mean_signal(i))^2 | MSS (error) |
|---|---|---|---|---|---|---|---|---|---|---|---|---|
| 0 | 0.184 | 0.354 | 0.185 | 0.2410 | -2.6866 | 8.570655 | 71.78369 | | 0.0032635 | 0.0128284 | 0.0031512 | 69,114165 |
| 1 | 2.836 | 3.030 | 2.280 | 2.716 | 0.6126 | 4.422551 | | | 0.0146431 | 0.0989010 | 0.1896549 | |
| 25 | 80.228 | 76.881 | 65.412 | 74.174 | 79.7942 | 31.589006 | | | 36.6635156 | 7.3275718 | 76.7724567 | |
| 50 | 162.479 | 173.562 | 164.512 | 166.851 | 162.2749 | 20.941082 | | | 19.1117561 | 45.0320553 | 5.4704220 | |
| 75 | 244.067 | 225.809 | 227.403 | 232.426 | 244.7556 | 152.008040 | | | 135.5023848 | 43.7865038 | 25.2346684 | |
| 100 | 321.946 | 351.20 | 333.582 | 335.5792 | 327.2364 | 69.603427 | | | 185.8669306 | 244.2929711 | 3.9863000 | |
| slope | 3.29923 | | | | | | | | | | | |
| intercept | -2.68659 | | | | | | | | | | | |
| n | 6 | | | | | | | | | | | |
| p | 3 | | | | | | | | | | | |
| DoF (numerator, n-2)= | | | 4 | | | | | | | | | |
| DoF (denominator, n*(p-1))= | | | 12 | | | | | | | | | |
| F_tab= | 3.26 | | | | | | | | | | | |
| F_calc= | 1.039 | | | | | | | | | | | |

**Table S7** Intraday precision (% CV) and accuracy (BIAS)

| | C teoret | C cal | BIAS | Average | BIAS | CV |
|---|---|---|---|---|---|---|
| PP1_1-1_001.lcd | 25 | 26.33 | 5.32 | 27.68 | 10.72 | 5.55 |
| PP2_1-1_002.lcd | 25 | 29.25 | 16.99 | | | |
| PP3_1-1_003.lcd | 25 | 26.37 | 5.46 | | | |
| PP4_1-1_004.lcd | 25 | 27.04 | 8.15 | | | |
| PP5_1-1_005.lcd | 25 | 29.42 | 17.68 | | | |
| | | | | | | |
| | **C teoret** | **C cal** | **BIAS** | **Average** | **BIAS** | **CV** |
| PP1_2-1_001.lcd | 75 | 74.79 | -0.28 | 72.09 | -3.88 | 3.95 |
| PP2_2-1_002.lcd | 75 | 69.26 | -7.66 | | | |
| PP3_2-1_003.lcd | 75 | 69.74 | -7.01 | | | |
| PP4_2-1_004.lcd | 75 | 71.26 | -4.98 | | | |
| PP5_2-1_005.lcd | 75 | 75.39 | 0.52 | | | |

**Table S8** Interday precision (% CV) and accuracy (BIAS)

| | C teoret | C cal | BIAS | Average | BIAS | CV |
|---|---|---|---|---|---|---|
| PP1_1-1_001.lcd | 25 | 26.33 | 5.32 | 27.68 | 10.72 | 5.55 |
| PP2_1-1_002.lcd | 25 | 29.25 | 16.99 | | | |
| PP3_1-1_003.lcd | 25 | 26.37 | 5.46 | | | |
| PP4_1-1_004.lcd | 25 | 27.04 | 8.15 | | | |
| PP5_1-1_005.lcd | 25 | 29.42 | 17.68 | | | |
| PP1_1-1_001.lcd | 25 | 25.68 | 2.71 | 26.29 | 5.17 | 3.56 |
| PP2_1-1_002.lcd | 25 | 25.09 | 0.38 | | | |
| PP3_1-1_003.lcd | 25 | 26.88 | 7.51 | | | |
| PP4_1-1_004.lcd | 25 | 27.45 | 9.81 | | | |
| PP5_1-1_005.lcd | 25 | 26.37 | 5.47 | | | |
| PP1_1-1_001.lcd | 25 | 26.81 | 7.26 | 27.14 | 8.55 | 7.37 |
| PP2_1-1_002.lcd | 25 | 26.52 | 6.08 | | | |
| PP3_1-1_003.lcd | 25 | 30.40 | 21.61 | | | |
| PP4_1-1_004.lcd | 25 | 27.01 | 8.03 | | | |
| PP5_1-1_005.lcd | 25 | 24.94 | -0.23 | | | |
| | **Average** | **27.04** | | | | |
| | BIAS | **8.15** | | | | |
| | CV | **5.84** | | | | |
| | **C teoret** | **C cal** | **BIAS** | **Average** | **BIAS** | **CV** |
| PP1_2-1_001.lcd | 75 | 74.79 | -0.28 | 72.09 | -3.88 | 3.95 |
| PP2_2-1_002.lcd | 75 | 69.26 | -7.66 | | | |
| PP3_2-1_003.lcd | 75 | 69.74 | -7.01 | | | |
| PP4_2-1_004.lcd | 75 | 71.26 | -4.98 | | | |
| PP5_2-1_005.lcd | 75 | 75.39 | 0.52 | | | |
| PP1_2-1_001.lcd | 75 | 74.19 | -1.09 | 71.42 | -4.77 | 4.15 |
| PP2_2-1_002.lcd | 75 | 68.95 | -8.06 | | | |
| PP3_2-1_003.lcd | 75 | 69.13 | -7.82 | | | |
| PP4_2-1_004.lcd | 75 | 69.75 | -7.00 | | | |
| PP5_2-1_005.lcd | 75 | 75.09 | 0.12 | | | |

| | | | | | | |
|---|---|---|---|---|---|---|
| PP1_2-1_001.lcd | 75 | 74.79 | -0.28 | 71.67 | -4.44 | 4.58 |
| PP2_2-1_002.lcd | 75 | 69.26 | -7.66 | | | |
| PP3_2-1_003.lcd | 75 | 69.44 | -7.42 | | | |
| PP4_2-1_004.lcd | 75 | 69.16 | -7.78 | | | |
| PP5_2-1_005.lcd | 75 | 75.70 | 0.93 | | | |
| | Mean | **71.42659** | | | | |
| | BIAS | **-4.36** | | | | |
| | CV | **5.08** | | | | |

**Table S9** ANOVA results for the interday precision study at concentration level 25 ng/mL

Summary

| *Groups* | *Count* | *Sum* | *Average* | *Variance* |
|---|---|---|---|---|
| Column 1 | 3 | 78.98998969 | 26.32999656 | 18.49 |
| Column 2 | 3 | 87.74182345 | 29.24727448 | 12.96 |
| Column 3 | 3 | 79.09551484 | 26.36517161 | 17.64 |
| Column 4 | 3 | 81.11133965 | 27.03711322 | 28.09 |
| Column 5 | 3 | 88.26311509 | 29.42103836 | 14.44 |

ANOVA

| *Source of variation* | *SS* | *df* | *MS* | *F* | *p-value* | *F crit* |
|---|---|---|---|---|---|---|
| Between Groups | 308.6577 | 4 | 7.089112655 | 0.386875827 | 0.813376308 | 3.478049691 |
| Within Groups | 257.2147 | 10 | 18.324 | | | |
| | | | | | | |
| Total | 565.8724 | 14 | | | | |

**Table S10.** ANOVA results for the intraday precision study at concentration level 25 ng/mL

Summary

| *Groups* | *Count* | *Sum* | *Average* | *Variance* |
|---|---|---|---|---|
| Column 1 | 5 | 138.4005942 | 27.68011885 | 2.363037552 |
| Column 2 | 5 | 131.4674723 | 26.29349446 | 1.775393768 |
| Column 3 | 5 | 135.6858044 | 27.13716088 | 3.997014495 |

ANOVA

| *Source of variation* | *SS* | *df* | *MS* | *F* | *p-value* | *F crit* |
|---|---|---|---|---|---|---|
| Between Groups | 4.88217265 | 2 | 2.441086327 | 0.900166893 | 0.4322649 | 3.88529383 |
| Within Groups | 32.5417833 | 12 | 2.711815272 | | | |
| | | | | | | |
| Total | 37.4239559 | 14 | | | | |

**Table S11.** ANOVA results for interday precision study at concentration level 75 ng/mL

| Summary | | | | | | |
|---|---|---|---|---|---|---|
| *Groups* | *Count* | *Sum* | *Average* | *Variance* | | |
| Column 1 | 3 | 224.3738 | 74.79127 | 38.44 | | |
| Column 2 | 3 | 207.772 | 69.25735 | 24.01 | | |
| Column 3 | 3 | 209.2212 | 69.74041 | 13.69 | | |
| Column 4 | 3 | 213.789 | 71.26301 | 29.16 | | |
| Column 5 | 3 | 226.1785 | 75.39283 | 18.49 | | |
| ANOVA | | | | | | |
| *Source of variation* | *SS* | *df* | *MS* | *F* | *p-value* | *F crit* |
| Between Groups | 97.30189 | 4 | 24.32547 | 0.98252977 | 0.459579828 | 3.478049691 |
| Within Groups | 247.58 | 10 | 24.758 | | | |
| | | | | | | |
| Total | 344.8819 | 14 | | | | |

**Table S12.** ANOVA results for intraday precision study at concentration level 75 ng/mL

Summary

| *Groups* | *Count* | *Sum* | *Average* | *Variance* |
|---|---|---|---|---|
| Column 1 | 5 | 360.444872 | 72.08897439 | 8.108490686 |
| Column 2 | 5 | 357.1107607 | 71.42215214 | 8.803633109 |
| Column 3 | 5 | 358.3432228 | 71.66864455 | 10.76238263 |

ANOVA

| *Source of variation* | *SS* | *df* | *MS* | *F* | *p-value* | *F crit* |
|---|---|---|---|---|---|---|
| Between Groups | 1.13681269 | 2 | 0.568406343 | 0.061616963 | 0.9405385 | 3.88529383 |
| Within Groups | 110.698026 | 12 | 9.224835474 | | | |
| | | | | | | |
| Total | 111.834838 | 14 | | | | |

**Table S13.** Summary of the accuracy study at $p < 0.05$

| | | *t*-value | *p*-value |
|---|---|---|---|
| Interday accuracy | 25 ng/mL | 1.72438 | 0.108319 |
| | 75 ng/mL | 1.72915 | 0.107439 |
| Intraday accuracy | 25 ng/mL | 1.15147 | 0.28734 |
| | 75 ng/mL | 1.37348 | 0.211976 |

**Table S14.** Recovery

| | C teoret | C cal | Average | BIAS | CV | Recovery |
|---|---|---|---|---|---|---|
| PP1_1-1_001.lcd | 25 | 26.63 | | 11.45 | 4.46 | 111.45 |
| PP2_1-1_002.lcd | 25 | 28.94 | 27.86 | | | |
| PP3_1-1_003.lcd | 25 | 26.97 | | | | |
| PP4_1-1_004.lcd | 25 | 27.34 | | | | |
| PP5_1-1_005.lcd | 25 | 29.42 | | | | |

The $t$-value is 1.68458. The $p$-value is 0.115912. The result is *not* significant at $p < 0.05$.

| | C teoret | C cal | Mean | BIAS | CV | |
|---|---|---|---|---|---|---|
| PP1_2-1_001.lcd | 75 | 71.15 | 70.57 | -5.90 | 2.68 | 94.10 |
| PP2_2-1_002.lcd | 75 | 68.95 | | | | |
| PP3_2-1_003.lcd | 75 | 70.04 | | | | |
| PP4_2-1_004.lcd | 75 | 69.14 | | | | |
| PP5_2-1_005.lcd | 75 | 73.57 | | | | |

The $t$-value is -1.51586. The $p$-value is 0.153488. The result is *not* significant at $p < 0.05$.
